# Strontium isotopes reveal weathering processes in lateritic covers in southern China with implications for paleogeographic reconstructions

**DOI:** 10.1371/journal.pone.0191780

**Published:** 2018-01-26

**Authors:** Xiao Wei, Shijie Wang, Hongbing Ji, Zhenhua Shi

**Affiliations:** 1 State Key Laboratory of Environmental Geochemistry, Institute of Geochemistry, Chinese Academy of Sciences, Guiyang, China; 2 College of Agriculture, Guizhou University, Guiyang, China; 3 Puding Karst Ecosystem Research Station, Chinese Academy of Sciences, Puding, China; 4 Civil and environmental engineering school, University of Science and Technology Beijing, Beijing, China; 5 Guizhou Geological Survey, Guiyang, China; Brigham Young University, UNITED STATES

## Abstract

The isotope ratios of Sr are useful tracers for studying parent material sources, weathering processes, and biogeochemical cycling. Mineralogical and geochemical investigations of two lateritic weathering covers, in an area close to the Tropic of Cancer (Guangxi Province, southern China), were undertaken to study the regional weathering processes and Sr isotopic sources. We found that weathering and decomposition of Rb- and Sr-bearing minerals change the Sr isotopic composition in weathering products (lateritic soils). Weathering of illite lowered the ^87^Sr/^86^Sr ratio whereas dissolving and leaching of carbonate minerals increased the ^87^Sr/^86^Sr ratio. An Fe nodular horizon is widely developed on the top of the weathering covers in the studied area and it differs from the lateritic soil horizon in mineral composition, construction, and elemental concentration. Furthermore, both Fe_2_O_3_ and P_2_O_5_ (concentrations) are negatively correlated with the ^87^Sr/^86^Sr ratios, suggesting fixation of apatite by Fe oxides is a controlling factor of the Sr isotopic composition in the Fe nodular horizon. The ^87^Sr/^86^Sr and Nb/Sr ratios imply the contents and proportions of Fe nodules and clay are critical in controlling the changes of Sr isotopic composition in the Fe nodular horizon. The two stages of the weathering process of carbonate rocks are revealed by the^87^Sr/^86^Sr versus Nb/Sr diagram. The ^87^Sr/^86^Sr and Rb/Sr ratios suggest that Sr isotopes in the weathering covers within the studied area are derived mainly from parent rock weathering and that the contributions from allothogenic Sr isotopes are limited. A comparison of Sr isotopic composition signatures in the weathering covers of the studied area and Guizhou Province provided insight into the Sr isotopic source and paleogeographic evolution of southern China. From the Permian to the Triassic, the continental fragment sources of the South China sedimentary basin changed significantly. In the Permian, Southern China presented the paleogeographic pattern that the north was higher (in elevation) than the south.

## Introduction

Weathering is a complex chemical, mechanical, and biological process that operates within various spheres of the land surface system, resulting in the disaggregation of rocks and minerals, pedogenesis, and the formation of weathering covers. The weathering process can change the geomorphologic signature of the land surface [1], control geochemical cycles of various elements [2], and provide nutrients necessary for life [3]. Weathering profiles are considered indicators of internal and external past conditions that may or may not persist. Therefore, studies of the weathering and pedogenesis of rocks and minerals are vital in many fields of geology and geochemistry [4–11].

Sr is an important trace constituent of various rock-forming minerals, and Sr isotopic geochemistry has been used extensively to address a wealth of earth science issues in rock weathering. For example, in natural water bodies, the specific mineral sources of dissolved Sr in catchment waters are studied via the ^87^Sr/^86^Sr ratio [12–18]. In addition, many attempts have been made to characterize groundwater–rock systems and to trace groundwater pathways in complex hydrogeological settings [19–24]. Recently, Krabbenhöft et al. [25] discussed the Sr isotope equilibrium between inputs and outputs of material during the last glacial maximum (10–30 ka before present) and in modern oceans. Sr isotopes have been used to study material sources of soils and terrestrial deposits [13, 26–27], nutrient cation cycling in ecosystems [28], and significant tectonism [29–30] because it is generally assumed that Sr isotopes are infrequently fractionated by near-surface chemical, physical, and biological processes [31]. However, each mineral is likely to have a distinct ^87^Sr/^86^Sr ratio [32], and the difference in weathering rates of minerals leads to the variation of Sr isotope composition in weathering products [12,33]. Therefore, Sr isotopes have been used as a tracer for explaining the weathering process [17, 34].

Fe–Mn nodules have strong adsorption capacity for many elements and they are distributed widely in the oceanic floor, lakes, streams, and soils [35–37]. Fe nodules in continental soils generally occur within the tropics, subtropics, Mediterranean climates, and even in temperate regions [37]. Soil Fe nodules are formed within the soil pore space because of seasonal changes in soil redox potential and pH [36, 38–39]. Both the formation and the growth of Fe nodules are also thought associated with bioturbation [40]. Adsorption and coprecipitation of Fe oxides are known to cause enrichment of many trace elements in soil Fe nodules [41]. Consequently, soil Fe nodules play an important role in studies addressing paleoclimate and paleoenvironment evolution, biogeochemical processes, and element enrichment mechanisms in supergene environments [36]. Several studies have published Sr isotopic compositions of Fe-Mn nodules in marine sediments [41–42]. However, current knowledge regarding Sr isotopic compositions of continental soil Fe nodules is limited.

**Fig 1:**
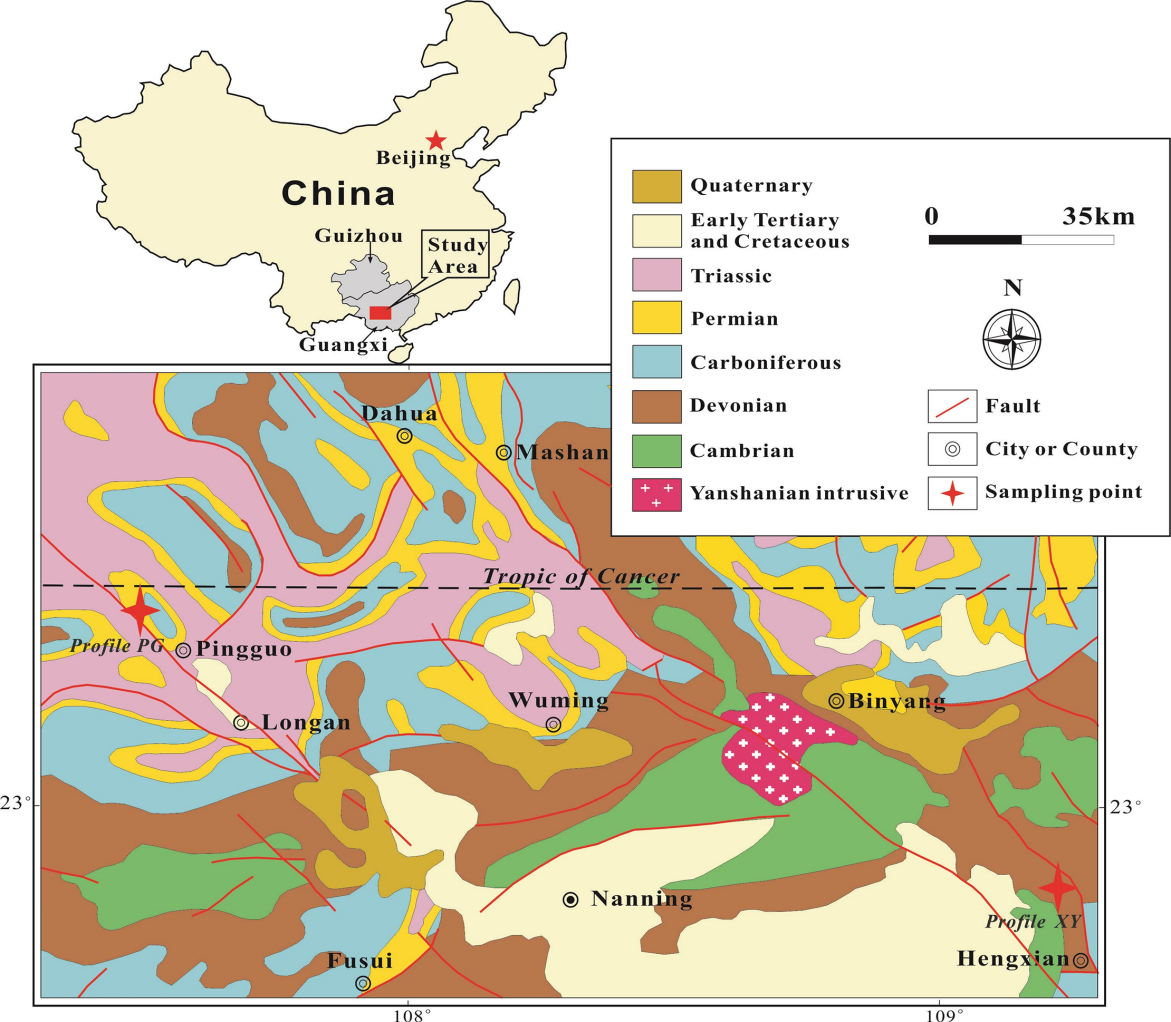
A map of the studied area illustrating locations, sampling sites, stratigraphic and structural characteristics.

Numerous weathering studies using Sr isotopes have been published; however, most have focused on igneous rocks [32, 43–46], while few have considered carbonate rocks, lateritic soils and soil Fe nodules [47]. In Guangxi Province, the widespread carbonate rocks were covered with thick lateritic soils because of the humid and mild monsoon climate. In the area of southern–central Guangxi Province, plenty of Fe nodules occur within the upper part of lateritic weathering covers, especially in the region around Binyang, Nanning, and Hengxian (Fig 1). Therefore, the natural setting facilitates the study of carbonate rocks, lateritic soils and Fe nodules. In this study, we investigated the Sr isotopic composition signatures of lateritic weathering covers by combining techniques of element geochemistry and mineralogy. The aims of this paper include: (1) the investigation of the mineralogical characteristics and ^87^Sr/^86^Sr ratio signatures of representative lateritic weathering covers; (2) the determination of mineralogical factors controlling Sr isotopic composition in different stages of rock weathering and pedogenesis; (3) the examination of the significance of ^87^Sr/^86^Sr ratio for evaluating weathering and pedogenesis processes of carbonate rocks; and (4) the evaluation of terrigenous Sr isotopic sources in the South China deposition basin during the Late Paleozoic.

## Setting

### Geomorphology and climate

Guangxi Province, located in southern China (Fig 1), has a mean elevation of 800 m and a wide range of geomorphic environments. The studied area is situated in the middle of Guangxi Province and it has a mean elevation of 50 m. The landforms are primarily karst plains and low hills that contrast with the mountainous region to the northwest and coastal plains to the south. The gentle terrain and hot and humid climate have promoted the formation and development of thick lateritic weathering covers. There is typically a loose Fe nodular horizon in the upper part of the weathering cover within this region, which is similar to the lateritic profiles in the equatorial zone described by Braun et al. [48].

The studied area lies near the Tropic of Cancer where the hot and humid tropical monsoon climate [49] and the alternation of wet (April–September) and dry (October–March) seasons are beneficial to the formation of Fe nodules [50]. The average annual temperature and average annual rainfall of the entire province are 21.5 ± 5°C and 1580 ± 300 mm/a, respectively. Evergreen broadleaf forest is the primary vegetation type within the studied area, which limits water and soil losses and induces strong evapotranspiration.

### Regional geology

Guangxi Province lies on the southern fringe of the Yangtze continental block. Multi-period and intensive tectonic movements have resulted in an outcrop pattern that includes various strata of differing ages (Fig 1). The oldest exposed strata in the studied area are Cambrian shale and sandstone. Between the Cambrian strata and overlying Devonian rocks, there is an angular unconformity, along which Ordovician and Silurian strata have been removed.

Devonian and Carboniferous rocks, distributed extensively within the studied area, are dominated by carbonate lithologies. Permian and Triassic successions are also dominated by carbonate rocks and they are in conformable contact with the underlying Carboniferous strata. The Yanshanian igneous body, formed during the Jurassic to Early Cretaceous, is exposed near Binyang County within the province and it comprises mainly biotite adamellite [49]. The Cretaceous and early Tertiary succession is dominated by red sandstone, separated from older rocks by another angular unconformity.

## Materials and methods

### Ethics statement

We state that no specific permissions were required for these locations/activities because the field work was not carried out on private land. Furthermore, in China, normal science studies are licensed and protected by the state.

### Sampling

To study the weathering and pedogenesis processes of carbonate rocks, as well as the formation and evolution mechanisms of Fe nodules within the tropics, two representative lateritic soil profiles in Guangxi Province were investigated. Profile XY is located in northern Hengxian County (22°51′6.6″N, 109°14′12.2″E) where the outcrops are mainly Devonian argillaceous limestone and calcareous shale. Profile PG is situated in western Pingguo County (23°24′20.5″N, 107°29′57.1″E), where the bedrock is Permian limestone (Fig 1). The different bedrock lithologies are responsible for the differences in mineralogical compositions and geochemical characteristics between profiles XY and PG.

**Fig 2:**
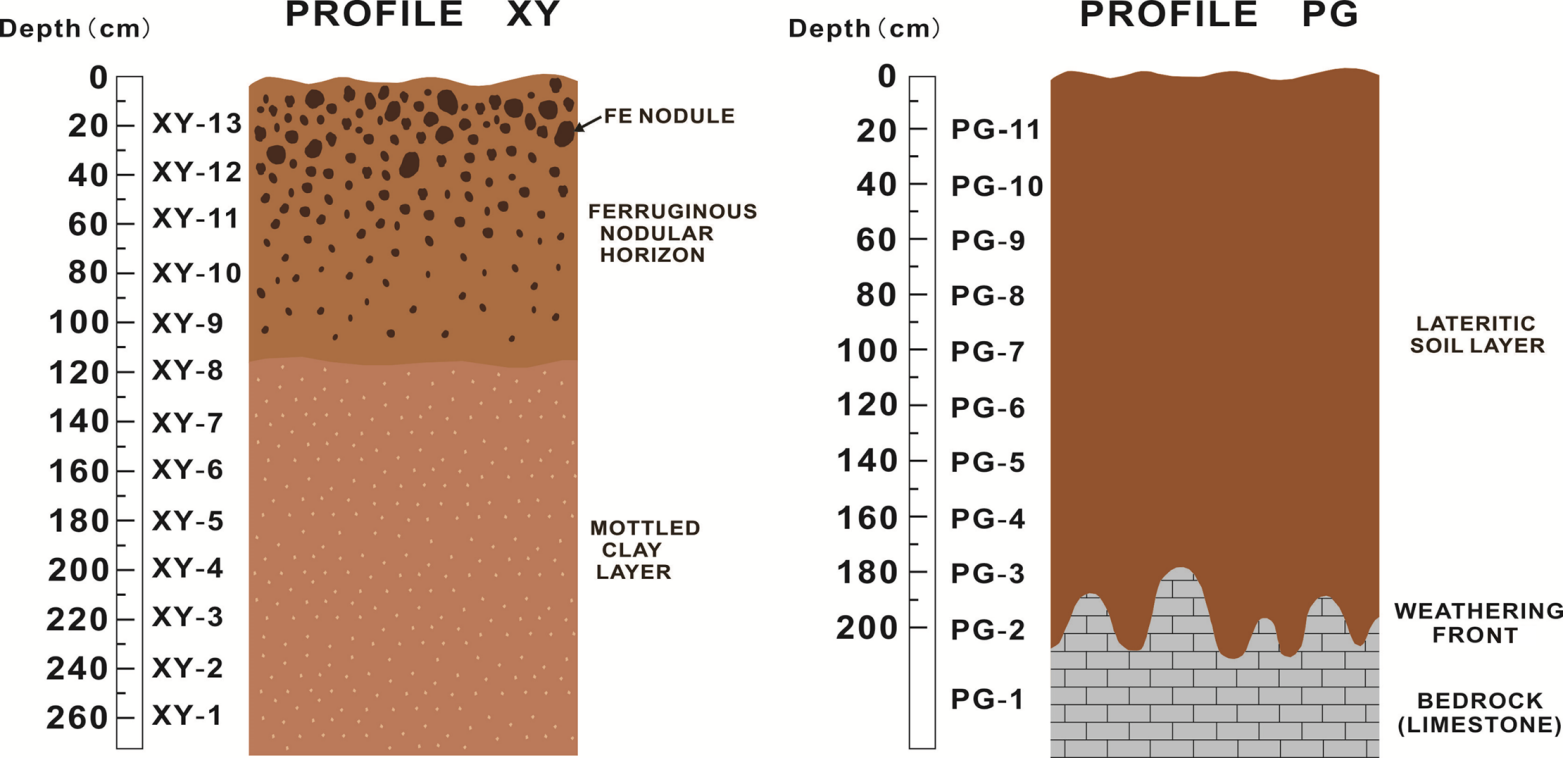
Schematic representation of studied weathering profiles showing the sectional structure and sampling depth.

Based on profile characteristics such as the structure, physical property, mineralogy, geochemistry, and weathering intensity, profile XY can be divided into two layers: an Fe nodular horizon and a mottled clay layer (Fig 2). The Fe nodular horizon contains abundant Fe nodules (sizes: 0.5–10.0 cm) distributed loosely within the lateritic soil. The large nodules (>1 cm) are composed of smaller nodules cemented by Fe oxides/hydroxides. Generally, the quantities and grain sizes of Fe nodules decrease with depth. The mottled clay layer consists of intermixed red and yellow clay. The profile was too thick to reach the bedrock and weathering front; however, the bedrock was assumed similar to the general geology within this area, i.e., Devonian argillaceous limestone. In contrast, profile PG is composed of homogeneous lateritic soil without obvious colored layering. The profile was created by excavators and the total thickness is about 2 m. The bedrock is the Permian Maokou Formation limestone and the weathering front is undulating.

Before sampling, the surface soil of each weathering profile (about 5–10 cm) was removed. Channel sampling was adopted to collect samples from bottom to top and each sample interval was 20 cm. The detailed sampling method follows [51]. Samples were collected and placed into sample sacks and transported to the laboratory within one week, where they were air dried for three weeks in the opened bags before processing.

### Analysis methods

The air-dried rock and soil samples used for the whole-rock geochemical analyses were ground in an agate mortar and sieved trough a 200 mesh (74 μm) light sieve.

X-ray diffraction (XRD) was used to determine the mineral composition, and the semi-quantitative calculation of the mineral phase was based on the lamellae spacing (d value) and X-ray relative intensity (I/I_1_). The experimental apparatus was a D/max–2000 model XRD diffractometer, including the instrument standard Cu Kα target, with settings of 40 Kv, 20 mA, and scanning scope for 2–60°.

For X-ray fluorescence (XRF) spectrometric analysis of the major elements, the powdered sample was mixed with lithium tetraborate flux and melted in a platinum crucible at a temperature of 1100°C. The fused samples were cooled into glass wafers and used for major element content testing using a Philips PW 2402 XRF spectrometer. The standard test errors for all major elements were <5%.

The CIA (chemical index of alteration) was used to evaluate the weathering extent quantitatively. CIA = Al_2_O_3_/(Al_2_O_3_ + CaO* + Na_2_O + K_2_O) × 100 [52], where oxide contents refer to molar contents and CaO* represents the CaO content in the silicate fraction of the test sample. The CaO contents in carbonate (calcite and dolomite) and phosphate (apatite) minerals are normally accomplished, calculated, and corrected by measured CO_2_ and P_2_O_5_ contents. The CaO* content is a corrected value, which is approximately equal to the surplus of the CaO content in the whole test samples minus the CaO content in the carbonate and phosphate minerals [53–54].

For trace element and Sr isotope analyses, powdered test samples were digested with a mixture of concentrated nitric and hydrofluoric acid (twice-distilled purification) in Teflon^®^ high–pressure digestion tanks at a temperature of 190°C for more than 48 h. The dissolved samples were split into two parts: one for trace element concentration analysis and the other for Sr isotopic composition testing. The trace element contents of the samples were measured by Inductively Coupled Plasma Mass Spectroscopy (ICP–MS) (Element I, Finnigan MAT Company) with Rh as the internal standard. The split for the Sr isotope testing was transformed into a hydrochloric acid medium by drying the distillate and adding hydrochloric acid (1.8–2.0 mol/L), repeatedly. The prepared solution was penetrated through cation exchange columns (AG50WX8, 200–400 mesh resin) to separate Sr from other ions. During the lessivation, 1.8–2.0 mol/L hydrochloric acid was used as an elution acid. Isotopic measurements were then performed by multicollector Thermal Ionization Mass Spectrometry (TIMS) (IsoProbe–T, U.K. GV Company). TIMS typically provides a value of the ^87^Sr/^86^Sr ratio of 0.710235 ± 0.000014 (2σ, n = 26) for the NBS987 Sr standard, and the measured isotope data are precise to 0.003% or better.

The major and trace element data of all samples were provided by the Beijing Research Institute of Uranium Geology, and the mineral and Sr isotopic composition testing was completed at the state Key Laboratory of Environmental Geochemistry, Institute of Geochemistry, Chinese Academy of Sciences.

In order to determine the bulk densities of the soils, the paraffin-coated clod method was used [55]. The pH values of the earth samples were determined with a pH meter. The solid-to-liquid ratio was 2.5:1.0, and the soak solution was ultra-pure water.

## Results

### Mineralogical composition

**Fig 3:**
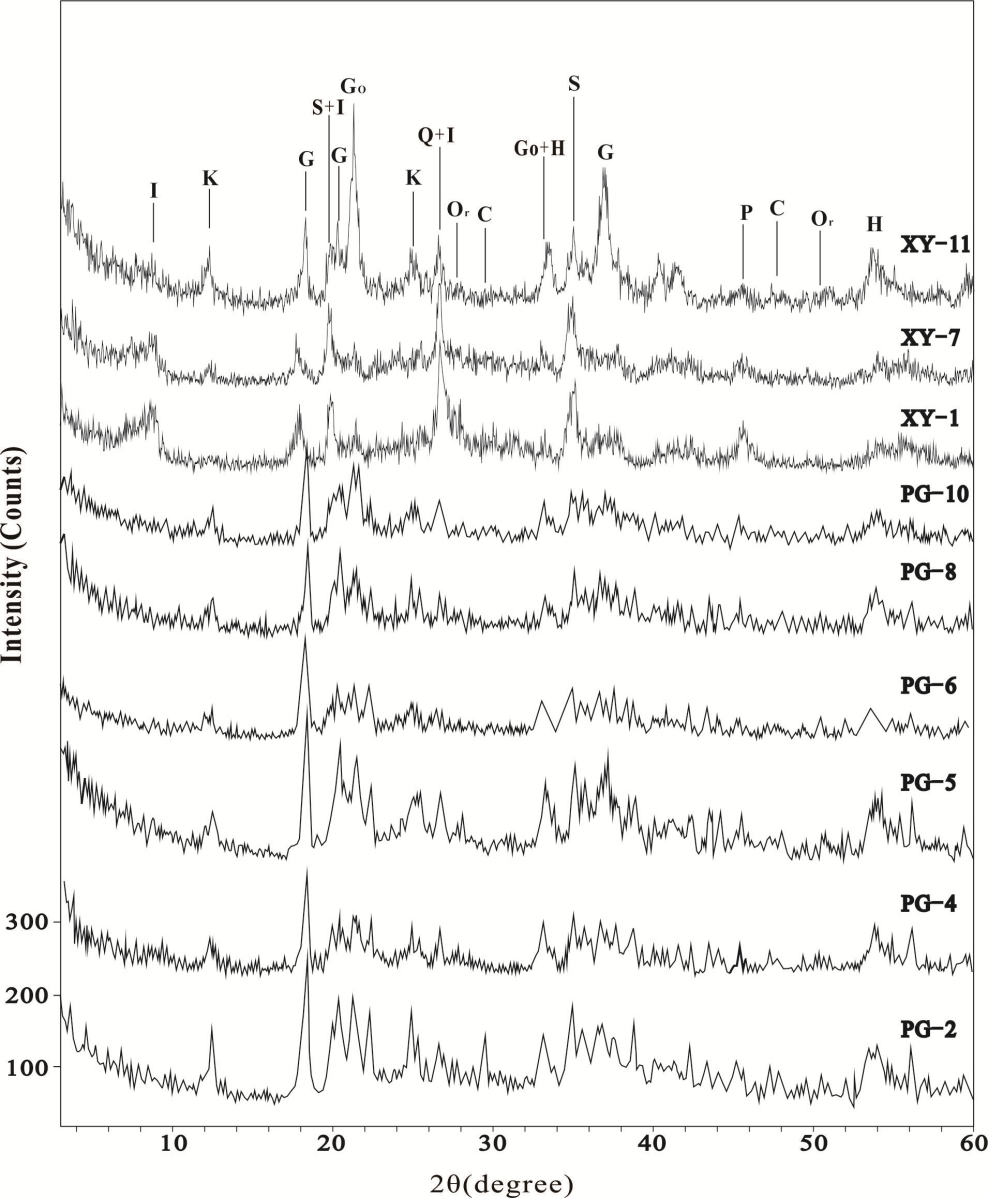
XRD patterns of selected Fe nodule and soil samples from profiles XY and PG. G–gibbsite, Go–goethite, H–hematite, I–illite, K–kaolinite, Or–orthoclase, P–pyrophyllite, Q–quartz, S–smectite, C–calcite.

In profile XY, the main minerals identified by XRD in the Fe nodular horizon (represented by sample XY–11) are iron oxide minerals (such as goethite and hematite), clay minerals (mainly kaolinite and small amounts of illite), and gibbsite (Fig 3). In the mottled clay layer (XY–1 and XY–7), the main minerals are illite and quartz with small amounts of smectite, calcite, plagioclase, and orthoclase. From the mottled clay layer to the Fe nodular horizon, the concentrations of illite and plagioclase decrease with increasing contents of kaolinite and iron oxides. In comparison, profile PG is characterized by high contents of kaolinite and gibbsite and low content of illite. The mineralogical compositions and concentrations in the samples of profile PG do not show particular variation, except the calcite content is higher in sample PG–2.

### Chemical composition

**Fig 4:**
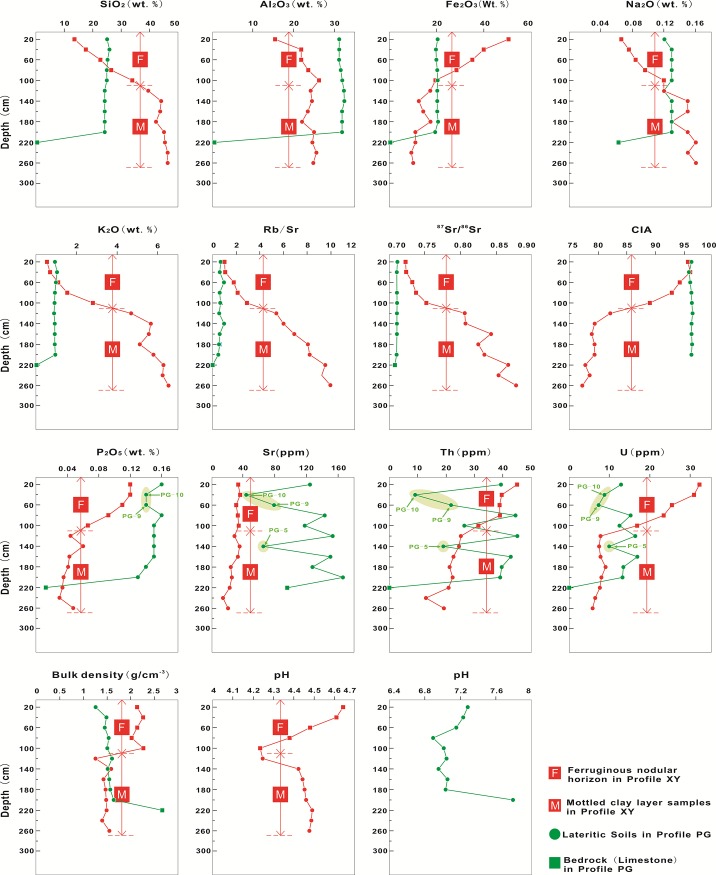
Distributions of selected major elements and changes of several relevant physicochemical parameters along with the vertical sampling depth.

The selected major and trace element concentrations, CIA values, and ^87^Sr/^86^Sr ratios of the bulk samples are listed in Tables 1 and 2, and the variation of several selected element concentrations and other geochemical parameters in the vertical direction are plotted in Fig 4. Because of the dilution effect of CaCO_3_, various elements in the bedrock sample (PG–1) of profile PG show very low contents but Sr remains relatively high. Concentrations of multiple major elements in profile PG have no significant changes with depth, whereas concentrations of several trace elements (Sr, Th, and U) show significant fluctuation. P_2_O_5_, Sr, Th, and U display obvious depleted contents in lateritic soil samples PG–5, PG–9, and PG–10. Concentrations of Fe_2_O_3_, P_2_O_5_, Sr, Th, and U in profile XY decrease with depth, and in the Fe nodular horizon, these elements are more enriched than in the soil layers. However, concentrations of other oxides (SiO_2_, Al_2_O_3_, Na_2_O, and K_2_O) show the opposite trend (Fig 4), which is interpreted as the dilution effect of iron [50].

**Table 1:** Selected major and trace element concentrations, ^87^Sr/^86^Sr ratios and physicochemical parameters of bulk samples of weathering profile XY in the study area.

Profile						PROFILE XY							
Sample	XY–1	XY–2	XY–3	XY–4	XY–5	XY–6	XY–7	XY–8	XY–9	XY–10	XY–11	XY–12	XY–13
Petrography	M ^*a*^	M ^*a*^	M ^*a*^	M ^*a*^	M ^*a*^	M ^*a*^	M ^*a*^	M ^*a*^	F ^*a*^	F ^*a*^	F ^*a*^	F ^*a*^	F ^*a*^
Bulk density (g/cm^-3^)	1.55	1.39	1.49	1.48	1.47	1.43	1.59	1.25	2.27 ^*b*^	2.03 ^*b*^	2.14 ^*b*^	2.26 ^*b*^	2.13 ^*b*^
pH	4.48	4.49	4.49	4.46	4.45	4.44	4.42	4.24	4.23 ^*c*^	4.38 ^*c*^	4.48 ^*c*^	4.61 ^*c*^	4.64 ^*c*^
CIA	77.23	78.49	77.73	79.35	79.33	78.87	79.34	82.08	89.00	92.79	94.23	96.06	95.63
SiO_2_/%	46.37	46.37	45.31	45.03	42.36	43.72	44.09	39.53	33.94	26.47	22.69	17.59	13.60
Al_2_O_3_/%	24.86	25.58	24.60	24.99	22.05	23.47	24.47	24.15	26.21	23.62	21.86	21.78	15.41
Fe_2_O_3_/%	10.02	9.44	10.97	11.00	17.50	14.36	12.49	17.39	19.49	28.44	35.13	39.96	50.36
CaO/%	0.06	0.06	0.09	0.07	0.06	0.07	0.08	0.05	0.11	0.11	0.12	0.10	0.08
P_2_O_5_/%	0.05	0.03	0.03	0.04	0.04	0.04	0.06	0.04	0.07	0.09	0.11	0.12	0.12
Na_2_O/%	0.16	0.15	0.16	0.15	0.13	0.15	0.15	0.12	0.12	0.096	0.084	0.076	0.066
K_2_O/%	6.53	6.25	6.27	5.78	5.11	5.58	5.66	4.69	2.81	1.55	1.11	0.71	0.55
Rb/×10^−6^	213.00	136.00	222.00	214.00	196.00	228.00	215.00	155.00	98.90	69.90	55.30	38.50	33.90
Sr/×10^−6^	21.30	14.70	23.30	26.10	24.30	33.10	35.70	28.80	33.90	33.10	31.00	36.70	33.60
Rb/Sr	10.00	9.25	9.53	8.20	8.07	6.89	6.02	5.38	2.92	2.11	1.78	1.05	1.01
Nb/×10^−6^	16.70	16.30	17.10	19.00	16.60	18.10	21.40	21.60	27.90	30.80	29.80	37.20	34.90
Nb /Sr	0.78	1.11	0.73	0.73	0.68	0.55	0.60	0.75	0.82	0.93	0.96	1.01	1.04
^**8**7^Sr/^86^Sr	0.878079	0.853491	0.867591	0.833849	0.825195	0.843111	0.807383	0.806676	0.752441	0.738346	0.732884	0.724301	0.723564

Note

*a* M, F refer to mottled clay layer and Fe nodular horizon, respectively.

*b* They are the bulk density of Fe nodules.

*c* They are the pH value of soils which surround the Fe nodules.

**Table 2:** Selected major and trace element concentrations, 87Sr/86Sr ratios and physicochemical parameters of bulk samples of weathering profile PG in the study area.

Profile						PROFILE PG					
Sample	PG–1	PG–2	PG–3	PG–4	PG–5	PG–6	PG–7	PG–8	PG–9	PG–10	PG–11
Petrography	L ^*a*^	S ^*a*^	S ^*a*^	S ^*a*^	S ^*a*^	S ^*a*^	S ^*a*^	S ^*a*^	S ^*a*^	S ^*a*^	S ^*a*^
Bulk density (g/cm^-3^)	2.67	1.64	1.57	1.55	1.51	1.61	1.50	1.53	1.45	1.49	1.26
pH	— ^*b*^	7.79	7.03	7.05	6.95	7.04	7.01	6.89	7.15	7.23	7.28
CIA	—	96.21	96.35	96.31	96.41	96.48	96.32	96.24	96.01	95.85	96.30
SiO_2_/%	0.70	24.04	24.21	24.21	24.20	24.30	24.85	24.90	25.17	25.93	24.99
Al_2_O_3_/%	0.54	31.80	31.89	31.89	32.51	32.37	31.90	31.53	31.20	31.19	31.30
Fe_2_O_3_/%	0.16	19.40	20.71	20.80	20.30	20.36	20.30	20.32	19.91	19.80	20.32
CaO/%	52.61	1.74	0.48	0.44	0.37	0.36	0.37	0.42	0.57	0.49	0.43
P_2_O_5_/%	0.01	0.13	0.14	0.15	0.15	0.15	0.15	0.16	0.14	0.14	0.16
Na_2_O/%	0.063	0.13	0.13	0.13	0.13	0.12	0.13	0.13	0.13	0.13	0.12
K_2_O/%	0.02	0.96	0.92	0.93	0.92	0.91	0.93	0.94	1.00	1.05	0.93
Rb/×10^−6^	—	81.20	77.20	91.40	66.00	86.30	79.30	88.80	76.20	27.00	85.20
Sr/×10^−6^	95.80	166.00	127.00	151.00	66.20	153.00	118.00	143.00	79.50	44.10	125.00
Rb/Sr	—	0.49	0.61	0.61	1.00	0.56	0.67	0.62	0.96	0.61	0.68
Nb/×10^−6^	0.05	58.60	63.60	73.70	71.00	73.60	61.70	58.60	53.50	52.70	51.50
Nb /Sr	0.00	0.35	0.50	0.49	1.07	0.48	0.52	0.41	0.67	1.20	0.41
^87^Sr/^86^Sr	0.707991	0.710465	—	0.711050	0.711050	0.711030	0.711052	0.711103	—	0.711059	0.711279

Note

*a* L and S refer to limestone and lateritic soil samples, respectively.

*b—*represents unanalyzed data or the content below the detection limit.

**Fig 5:**
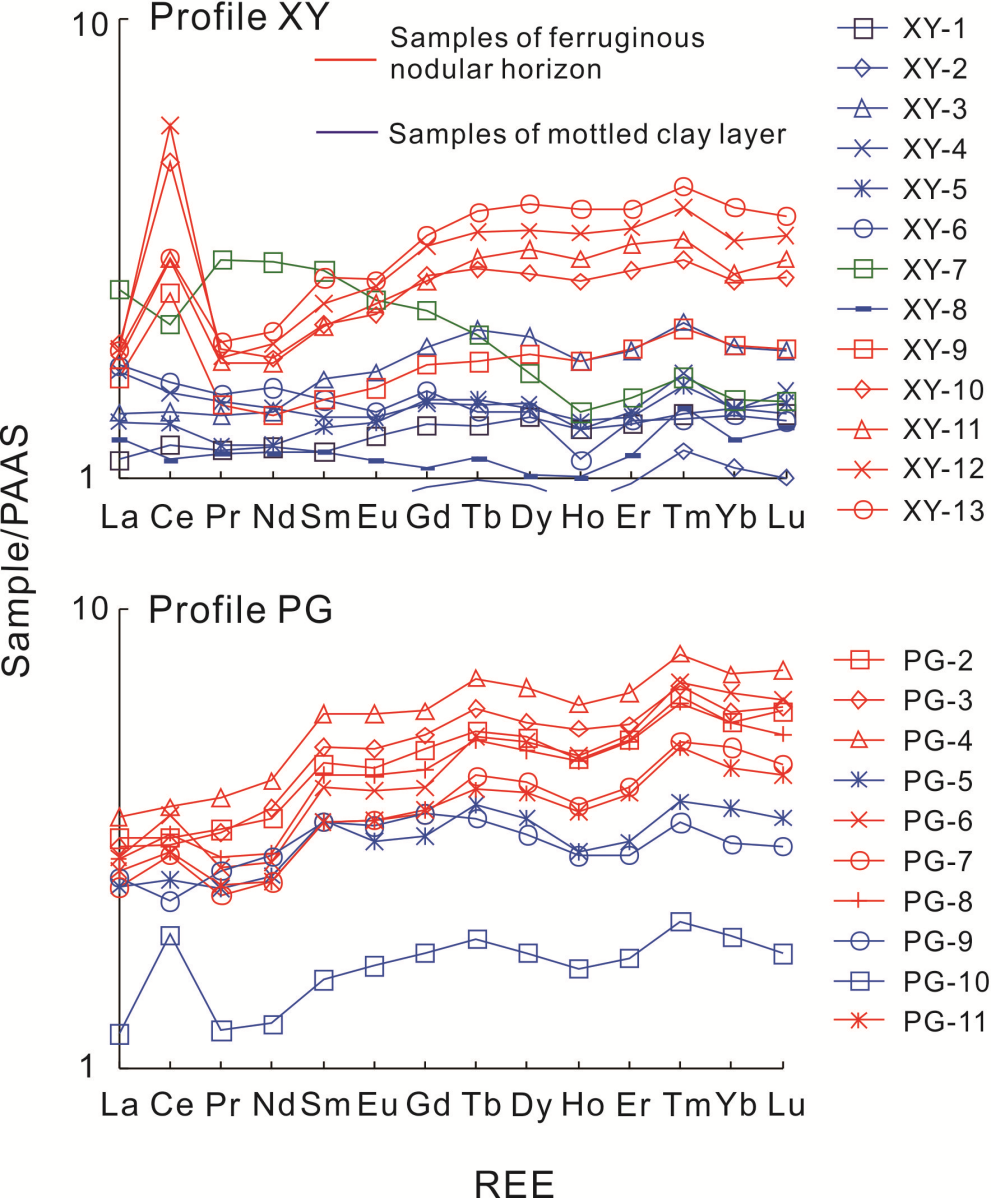
PAAS (Post–Archean Australian Shale) normalized REE distribution patterns of profiles XY and PG.

The rare earth element (REE) contents and relevant parameters are listed in Tables 3 and 4, and PAAS (Post-Archean Australian Shale) normalized REE distribution patterns [56] are shown in Fig 5. In profile XY, the test data show that the total REE content (ΣREE) in the nodules (366.57–696.08 ppm) is higher than in the mottled clay (162.86–296.28 ppm, except in sample XY–7: 445.81 ppm). The obvious REE fractionations between the Fe nodular horizon and mottled clay layer are observed in Fig 5. Compared with the mottled clay, the Fe nodules show enrichment of middle and heavy REEs (MREEs and HREEs, respectively) and loss of light REEs (LREEs). Ce anomalies in the whole profile are not obvious (δCe rangs from 0.89 to 1.01). The REEs in sample XY–7 yield evident fractionations. The higher ΣREE and LREE contents indicate that this horizon is the zone of REE enrichment. In profile PG, compared with the bed rock (PG-1) (ΣREE is 1.07), soils presents significant REE concentration (ΣREE ranges from 296.06 to 759.81 ppm). Sample PG-1 shows obvious negative Ce anomaly (δCe is 0.68), whereas soil samples have no distinct Ce anomalies (δCe rangs from 0.87to 1.27), except PG-10 (δCe is 1.63). Samples PG–5, PG–9, and PG–10 have depleted ΣREE (491.14, 483.05, and 296.06 ppm) compared with the other lateritic soil samples (528.48–759.81 ppm). In addition, samples PG–5, PG–9, and PG–10 show distinct depleted MREEs and HREEs in Fig 5.

**Table 3:** Rare earth element concentrations and relevant parameters of bulk samples in weathering profiles XY in the study area.

Profile						PROFILE XY							
Sample	XY–1	XY–2	XY–3	XY–4	XY–5	XY–6	XY–7	XY–8	XY–9	XY–10	XY–11	XY–12	XY–13
La	42.00	31.50	53.10	65.40	50.80	67.20	98.60	46.70	63.40	74.90	69.10	73.40	72.80
Ce	94.40	82.70	111.00	122.00	105.00	129.00	173.00	87.40	202.00	389.00	239.00	466.00	241.00
Pr	10.20	7.25	12.10	13.00	10.40	13.50	26.50	10.00	12.80	16.90	15.80	16.20	17.50
Nd	38.80	28.20	46.10	47.20	39.20	52.10	97.9	37.70	45.60	60.50	58.90	64.90	69.30
Sm	6.35	4.94	9.11	7.53	7.20	8.22	15.70	6.37	8.25	12.00	11.90	13.30	15.20
Eu	1.34	0.97	1.84	1.47	1.43	1.51	2.65	1.18	1.70	2.45	2.60	2.83	2.92
Gd	6.11	4.47	9.03	6.77	6.91	7.25	10.80	4.91	8.25	12.90	12.50	14.90	15.80
Tb	1.01	0.767	1.63	1.12	1.15	1.08	1.59	0.86	1.39	2.21	2.33	2.66	2.94
Dy	6.39	4.52	9.52	6.79	6.64	6.52	7.93	4.76	8.73	13.10	14.70	16.20	18.50
Ho	1.27	0.88	1.78	1.27	1.32	1.09	1.38	1.00	1.79	2.66	2.97	3.39	3.83
Er	3.75	2.79	5.41	3.98	3.87	3.82	4.27	3.21	5.47	8.08	9.19	10.00	11.00
Tm	0.56	0.47	0.89	0.69	0.65	0.55	0.67	0.58	0.86	1.21	1.34	1.57	1.75
Yb	4.01	2.97	5.46	3.97	4.02	3.87	4.18	3.43	5.50	7.56	7.86	9.27	11.00
Lu	0.60	0.44	0.82	0.67	0.63	0.58	0.64	0.56	0.83	1.18	1.30	1.46	1.61
δEu^a^	1.05	1.11	1.01	0.97	1.05	0.99	0.78	0.93	1.64	2.52	1.67	3.12	1.56
δCe^a^	1.01	0.97	0.96	0.97	0.95	0.92	0.96	0.99	0.97	0.93	1.00	0.95	0.89
ΣREE	216.79	162.86	267.79	281.86	239.22	296.28	445.81	208.66	366.57	604.65	449.49	696.08	485.15

Note

*a* represent PAAS (Post–Archean Australian Shale) normalized values [56].

**Table 4:** Rare earth element concentrations and relevant parameters of bulk samples in weathering profiles PG in the study area.

Profile						PROFILE PG					
Sample	PG–1	PG–2	PG–3	PG–4	PG–5	PG–6	PG–7	PG–8	PG–9	PG–10	PG–11
La	0.29	121.00	116.00	135.00	95.30	110.00	94.80	109.00	99.40	45.50	103.00
Ce	0.37	254.00	244.00	295.00	205.00	284.00	232.00	258.00	184.00	156.00	235.00
Pr	0.06	29.30	28.80	34.20	21.80	24.20	21.10	25.40	23.80	10.70	22.10
Nd	0.19	116.00	122.00	140.00	87.00	92.70	84.50	97.30	96.00	41.50	84.50
Sm	0.02	25.60	27.80	32.80	19.20	22.60	19.10	24.20	19.10	8.67	19.10
Eu	0.01	4.87	5.37	6.40	3.37	4.34	3.76	4.70	3.64	1.81	3.74
Gd	0.03	23.00	24.70	27.90	14.90	19.00	16.70	20.80	16.70	8.35	17.00
Tb	0.01	4.19	4.69	5.47	2.91	4.08	3.35	4.00	2.71	1.48	3.13
Dy	0.04	24.60	26.60	31.50	16.40	24.10	19.60	23.00	15.10	8.35	18.60
Ho	0.01	4.67	5.43	6.13	2.93	4.75	3.70	4.60	2.88	1.64	3.58
Er	0.03	14.80	15.90	18.70	8.87	15.20	11.70	14.60	8.31	4.96	11.30
Tm	0.01	2.60	2.76	3.23	1.54	2.81	2.08	2.53	1.39	0.85	2.02
Yb	0.03	16.00	16.80	20.30	10.40	18.50	14.10	16.00	8.70	5.48	12.70
Lu	—^b^	2.59	2.64	3.18	1.52	2.74	1.99	2.31	1.32	0.77	1.88
δEu^a^	1.39	0.95	0.96	1.00	0.94	0.99	0.99	0.99	0.96	1.00	0.98
δCe^a^	0.68	0.98	0.97	1.00	1.04	1.27	1.20	1.13	0.87	1.63	1.14
ΣREE	1.07	643.22	643.49	759.81	491.14	629.02	528.48	606.44	483.05	296.06	537.65

Note

*a* represent PAAS (Post–Archean Australian Shale) normalized values [56].

*b—*represents unanalyzed data or the content below the detection limit.

### Sr isotope signature and CIA (chemical index of alteration)

The variations in range of CIA values and ^87^Sr/^86^Sr and Rb/Sr ratios in the samples of profile XY are larger (CIA = 77.23–96.06, ^87^Sr/^86^Sr = 0.723564–0.878079, Rb/Sr = 1.01–10.00). By contrast, those parameters in the samples of profile PG do not change significantly (CIA = 95.85–96.48, ^87^Sr/^86^Sr = 0.710465–0.711279, Rb/Sr = 0.49–0.95) (Tables 1 and 2). In comparison with profile PG, profile XY has higher ^87^Sr/^86^Sr and Rb/Sr ratios and lower CIA values (Fig 4).

The CIA values of profile XY decrease with depth (Fig 4). In addition, samples in the Fe nodular horizon (depth: 100 cm) of profile XY show a higher degree of weathering than the mottled clay layer (below 120 cm). The CIA values of profiles PG are higher and there are no significant changes in CIA as the sampling depth increases. The ^87^Sr/^86^Sr and Rb/Sr ratios of profile XY increase downward. The ^87^Sr/^86^Sr ratios in profile PG are nearly constant, although the Rb/Sr ratios of samples at 60 and 140 cm in profile PG have a slight increase.

## Discussion

### Changes of Sr isotopic composition in weathering covers

Sr isotopes are infrequently fractionated by near-surface chemical, physical, and biological processes [31]. However, weathering and decomposition of specific Sr-bearing minerals could lead to the modification of the Sr isotopic composition [32, 57–62]. The ^87^Sr/^86^Sr ratios of profile XY decrease upwards with the intensification of weathering, while those of profile PG remain nearly constant (Fig 4). The reasons and controlling factors for the changes in the Sr isotopic composition of the two profiles are discussed below.

**Fig 6:**
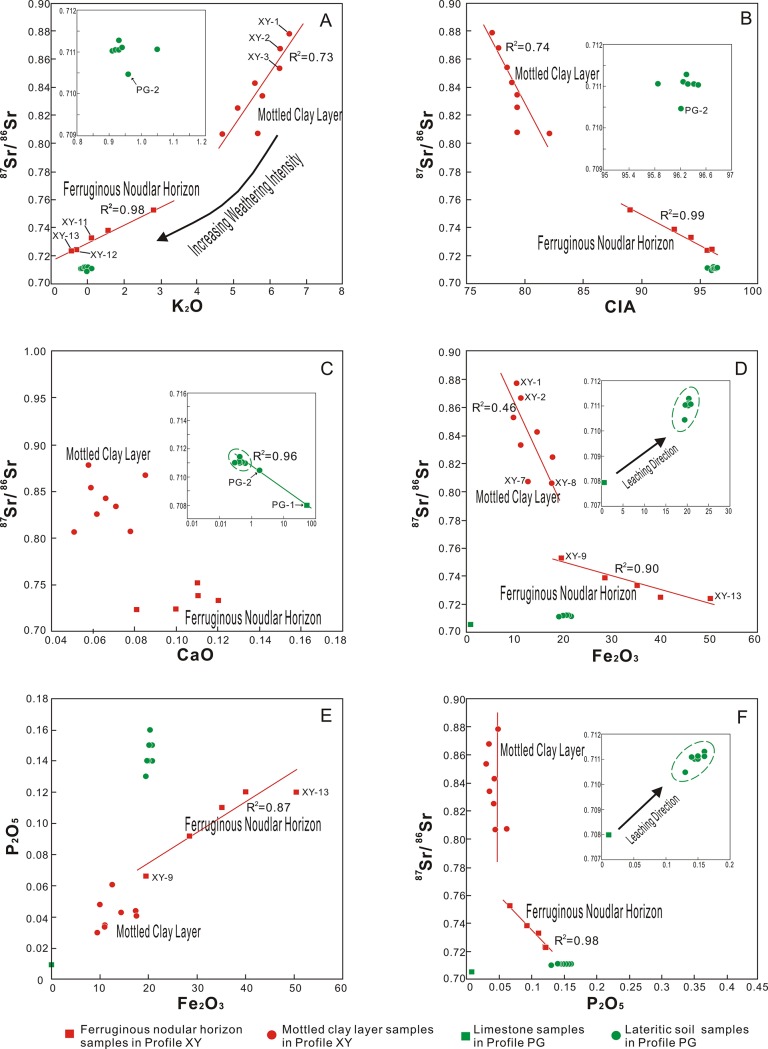
**Distributions of** ^**87**^**Sr/**^**86**^**Sr ratio with (A) K**_**2**_**O, (B) CIA value, (C) CaO, (D) Fe**_**2**_**O**_**3**_**, and (F) P**_**2**_**O**_**5**_ **and relationships between (E) Fe**_**2**_**O**_**3**_ **and P**_**2**_**O**_**5**_. Significant correlations are shown in the plots. CaO refers to the total content.

XRD data show that from the bottom to top of profile XY, illite decreases as kaolinite increases. It indicates that illite is weathered and transformed into kaolinite with increasing weathering intensity (Fig 3). Meanwhile, XRD data also indicate the main K-bearing mineral in profile XY is illite (Fig 3). Therefore, the upward decrease of K_2_O concentrations in profile XY also confirms the weathering and decomposition of illite (Fig 4). In addition, the ^87^Sr/^86^Sr ratios show obvious positive correlations with K_2_O concentrations (R^2^ = 0.98 in the Fe nodular horizon and R^2^ = 0.73 in the mottled clay layer) and negative correlations with CIA value (R^2^ = 0.99 in the Fe nodular horizon and R^2^ = 0.74 in the mottled clay layer) in profile XY (Fig 6A and 6B). It suggests a strong link between the weathering and decomposition of illite and changes of the ^87^Sr/^86^Sr ratios in profile XY.

K-bearing minerals usually contain abundant Rb because of the similar ionic radii and equal electrovalence of K and Rb. Accordingly, the ^87^Sr/^86^Sr ratios in K-bearing minerals such as biotite, K-feldspar, and illite are usually very high because ^87^Sr is derived from ^87^Rb by beta decay. Therefore, decomposition of K-bearing minerals in rocks might lead to decreased ^87^Sr/^86^Sr ratios in the residual weathering products relative to their parent rocks. [61, 63–65]. In summary, the appearance of illite in profile XY is the key factor behind the higher ^87^Sr/^86^Sr ratios in the weathering cover. Furthermore, the weathering and decomposition of illite is a critical controlling factor for ^87^Sr/^86^Sr ratios. However, compared with profile XY, the samples in profile PG have lower K_2_O concentrations and no significant correlation between K_2_O concentrations and ^87^Sr/^86^Sr ratios (Figs 4 and 6A).

Liu et al. [62] have done a leaching experiment of limestone in Guizhou province. The result shows that Sr isotopes in carbonate rock have two distinct sources: one part derives from carbonate minerals with higher Sr concentration and a lower ^87^Sr/^86^Sr ratio, and the other part is provided by relatively stable silicate minerals with lower Sr concentration and a higher ^87^Sr/^86^Sr ratio. In a hot and humid environment, Ca and Sr in carbonate minerals are easily leached and migrated, resulting in an increase of the ^87^Sr/^86^Sr ratios in the residual soil [62, 66]. Profile PG is a typical terra rossa profile which presents slight alkaline environment (pH values of profile PG range from 6.89–7.79 (Table 2)) and relatively high CaO content and therefore, the significant negative correlation (R^2^ = 0.96) between the CaO contents and ^87^Sr/^86^Sr ratios reflects the weathering and leaching processes of carbonate minerals (Fig 6C). On the contrary, profile XY is an acid lateritic weathering crust (pH values of profile XY range from 4.24–4.64 (Table 1)) which presents a complete decalcification hence CaO contents and ^87^Sr/^86^Sr ratios do not present any significant linear relations. However, compared with the mottled clay layer, the Fe nodular horizon has higher CaO contents and lower ^87^Sr/^86^Sr ratios, implying the lower ^87^Sr/^86^Sr ratios are possibly associated with Ca-bearing minerals. To a large extent, the Ca-bearing mineral is not carbonate, because of the strong eluviation and acid environment. The following discussion suggests the Ca-bearing mineral might possibly be apatite.

The Fe_2_O_3_ contents and ^87^Sr/^86^Sr ratios in profile XY have significant negative correlations, especially in the Fe nodular horizon (R^2^ = 0.90) (Fig 6D). It indicates that the Fe_2_O_3_ contents have certain effects on controlling the Sr isotopic composition, particularly for the Fe nodular horizon. Although it has been proven that iron oxides/hydroxides have strong adsorption ability for Sr [67], the influence of the adsorption and coprecipitation of Fe oxides/hydroxides on Sr isotopic fractionation appears less than likely because Sr isotopes are infrequently fractionated by near-surface chemical, physical, and biological processes [31]. Based on studies of Fe nodules in weathered crust formed by granodioritic gneisses in the Mysore Plateau of southern India, Tripathi and Rajamani [36] proposed that discrete amorphous Mn phases (e.g., todorokite [(Ca, Na, K) _0.3–0.5_ Mn (IV or III), Mg] _6_ O_12_ (3–4.5) H_2_O) concentrate trace elements including Sr. Todorokite is a mineral bearing K, Rb, and Sr and therefore, the content of todorokite in Fe nodules possibly influences the variation of the Sr isotope; however, discrete amorphous Mn phases were not observed in this study.

The result shows that weathering and decomposition of illite is one possible controlling factor of the Sr isotopic composition in the Fe nodular horizon. This is because the K_2_O concentration decreases with the increase of weathering intensity, and a significant positive correlation between K_2_O and the ^87^Sr/^86^Sr ratio is present in the Fe nodular horizon (R^2^ = 0.98) (Fig 6A). However, the positive correlation in the Fe nodular horizon appears to differ from that in the mottled clay layer (slopes of the two trend lines are different) (Fig 6A), which implies there is another factor controlling the Sr isotopic composition in the Fe nodular horizon or the mottled clay layer.

A relative accumulation of P_2_O_5_ and a significant linear relationship between P_2_O_5_ and Fe_2_O_3_ are found in the Fe nodular horizon (Fig 6E). This indicates that the Fe nodular horizon has strong adsorption and fixation to phosphate minerals [68–72]. According to the PAAS normalized REE pattern (Fig 5), compared with the mottled clay, the Fe nodules are characterized by high ΣREE and Sm_N_/Nd_N_ ratio (N represents PAAS normalized), MREE and HREE enrichment, and depleted LREE (except Ce). The concentrations of P_2_O_5_, Sr, Th, and U are higher in the Fe nodular horizon than in the mottled clay layer. Generally, MREE-enriched apatite contains abundant REEs, Th, U, and Sr and the weathering of apatite might cause strong REE fractionation [44, 73–81]. Consequently, apatite can be interpreted as the main phosphate phase in Fe nodules, although phosphate minerals were not detected by the XRD analysis. This is probably because phosphate mineral abundances are low in the study profiles [82] but their existence is verified by geochemical data (Tables 1 and 2).

Apatite is an important common mineral in various rocks (including igneous, metamorphic, and sedimentary rocks), sediments, and alluvial and residual deposits. Primary apatite is generally considered a type of Rb-free mineral with no radiogenic ^87^Sr [80], whereas secondary apatite might borrow radiogenic ^87^Sr from the ambient diagenetic fluid [80, 83], which means apatite generally has a lower ^87^Sr/^86^Sr ratio. A significant negative correlation between the P_2_O_5_ contents and ^87^Sr/^86^Sr ratios (R^2^ = 0.98) is observed in the Fe nodular horizon in profile XY (Fig 6F), which suggests that apatite fixed by Fe oxides is another mineralogical controlling factor of the Sr isotopic composition in the Fe nodular horizon.

### Significance of Sr isotopes for evaluating weathering components and processes

In a typical geochemical weathering process for igneous rocks, Sr isotope variation is commonly associated with the leaching loss of Sr. Niobium (Nb) is an immobile element and hence, the concentration of Nb in soil solution is very low and the Nb/Sr ratio approaches zero. The concentration of Nb and the Nb/Sr ratio in soils is expected to increase as the degree of weathering increases. For this reason, ^87^Sr/^86^Sr versus Nb/Sr diagrams are generally used to evaluate the active Sr endmembers and to reveal the evolutionary processes of weathering profiles [32, 66, 84].

**Fig 7:**
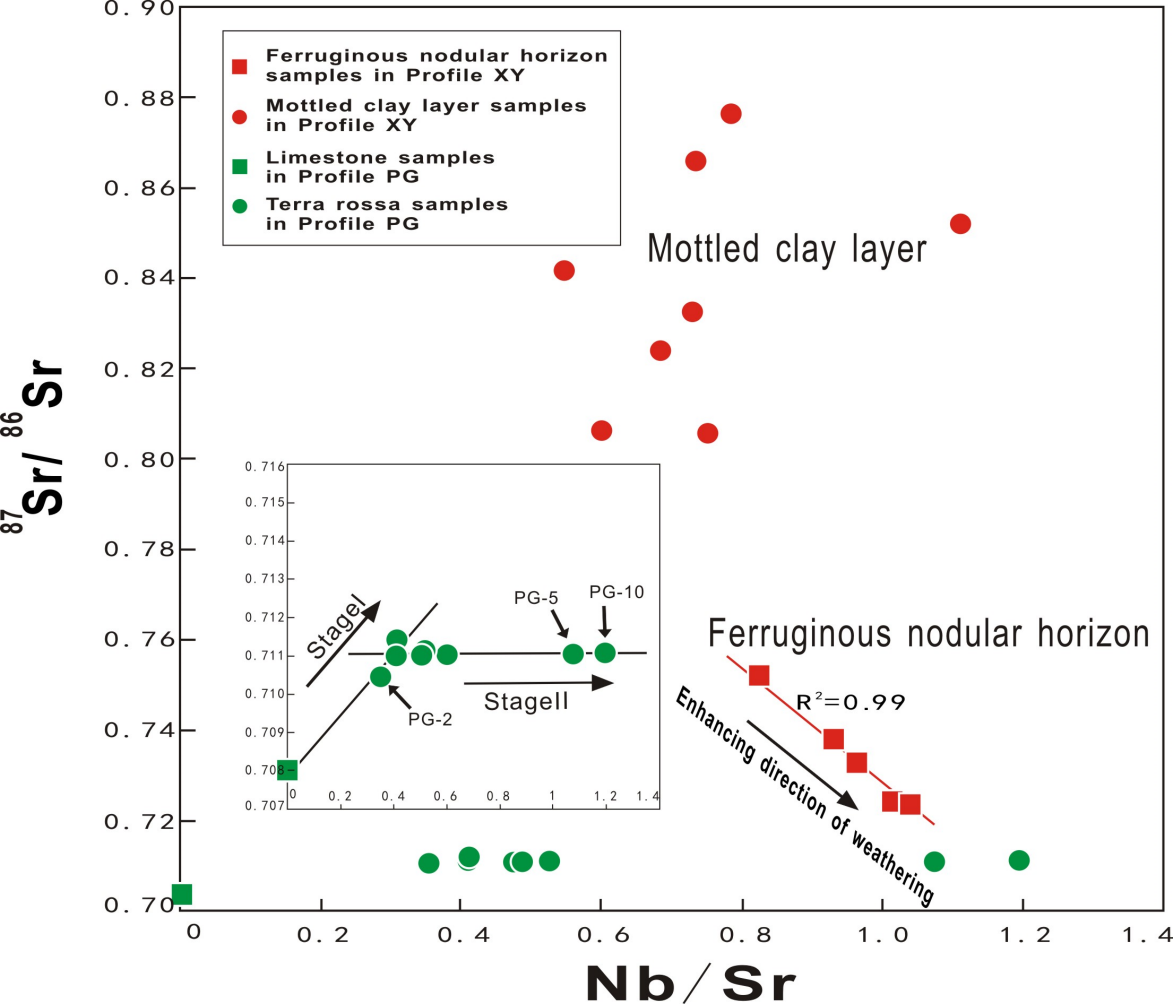
^87^Sr/^86^Sr ratio trends as function of Nb/Sr ratio in profiles XY and PG.

In profile XY, compared with the Fe nodular horizon, the mottled clay layer shows higher ^87^Sr/^86^Sr and lower Nb/Sr ratios (Fig 7). This illustrates the absolute Sr concentration decreases with increasing weathering intensity. The change of the ^87^Sr/^86^Sr ratio is associated mainly with the weathering and decomposition of illite; hence, the ^87^Sr/^86^Sr and Nb/Sr ratios do not show significant correlations in the mottled clay layer. However, the significant negative correlation (R^2^ = 0.99) in the Fe nodular horizon is a geochemical response of the proportions of Fe nodules and clay. In the Fe nodular horizon, increasing weathering intensity causes the content of Fe nodules to increase, whereas the clay content decreases (Fig 2). The decreased clay content results in decreased Sr concentrations and low Nb/Sr ratios. Meanwhile, the increase of Fe nodules benefits the fixation of apatite, which results in the decreased ^87^Sr/^86^Sr ratios in the Fe nodular horizon.

In Fig 7, the weathering front sample (PG–2) of profile PG is located between samples of bedrock and lateritic soil, implying binary mixing of two different Sr isotopic endmembers. A leaching experiment confirmed two distinct Sr isotopic sources in the carbonate rock: the carbonate and silicate mineral endmembers [62]. The carbonate endmember largely represents the Sr isotopic signature of seawater in the diagenesis period, whereas the silicate endmember is a label of the Sr isotopic composition of fragments from the weathering of continental rock. Because of the observably different rates of weathering between carbonate and silicate minerals, the weathering process of carbonate rocks can be divided into two stages [8]: Stage I representing the leaching process of carbonate minerals and stage II representing the weathering process of saprolite (Fig 7).

Fresh bedrock commonly represents the carbonate endmember with a relatively lower ^87^Sr/^86^Sr ratio and higher Sr content, because carbonate minerals take a dominant position over silicate impurities in the contents of carbonate rocks. Carbonate minerals are weathered preferentially under warm and humid conditions and thus, the silicate endmember with a higher ^87^Sr/^86^Sr ratio controls the Sr isotopic composition in saprolite. Therefore, Stage I presents a trend of increase in the ^87^Sr/^86^Sr ratio and a reduction in Sr content (Fig 7). In stage II, carbonates are exhausted (CaO concentration usually <0.5 wt.%) and silicate components constitute the main body of the regolith. During this process, silicate minerals suffer the effects of long periods of strong weathering and they transform into more stable minerals, such as kaolinite and gibbsite. Samples in the regolith of profile PG are distributed along a line that nearly parallels the Nb/Sr axis (Fig 7). However, samples PG–5 and PG–10 show higher Nb/Sr ratios, which might possibly be due to lower apatite contents (Figs 4 and 5). Results indicate that the changes in Sr concentration do not yield obvious alteration in the ^87^Sr/^86^Sr ratios. The constant ^87^Sr/^86^Sr ratios suggest that several main Sr-bearing carbonate minerals have been completely weathered and the residual Sr in the regolith might possibly have a similar ^87^Sr/^86^Sr ratio.

### Implications of Sr isotopes for material source tracing and paleogeographic reconstruction

**Fig 8:**
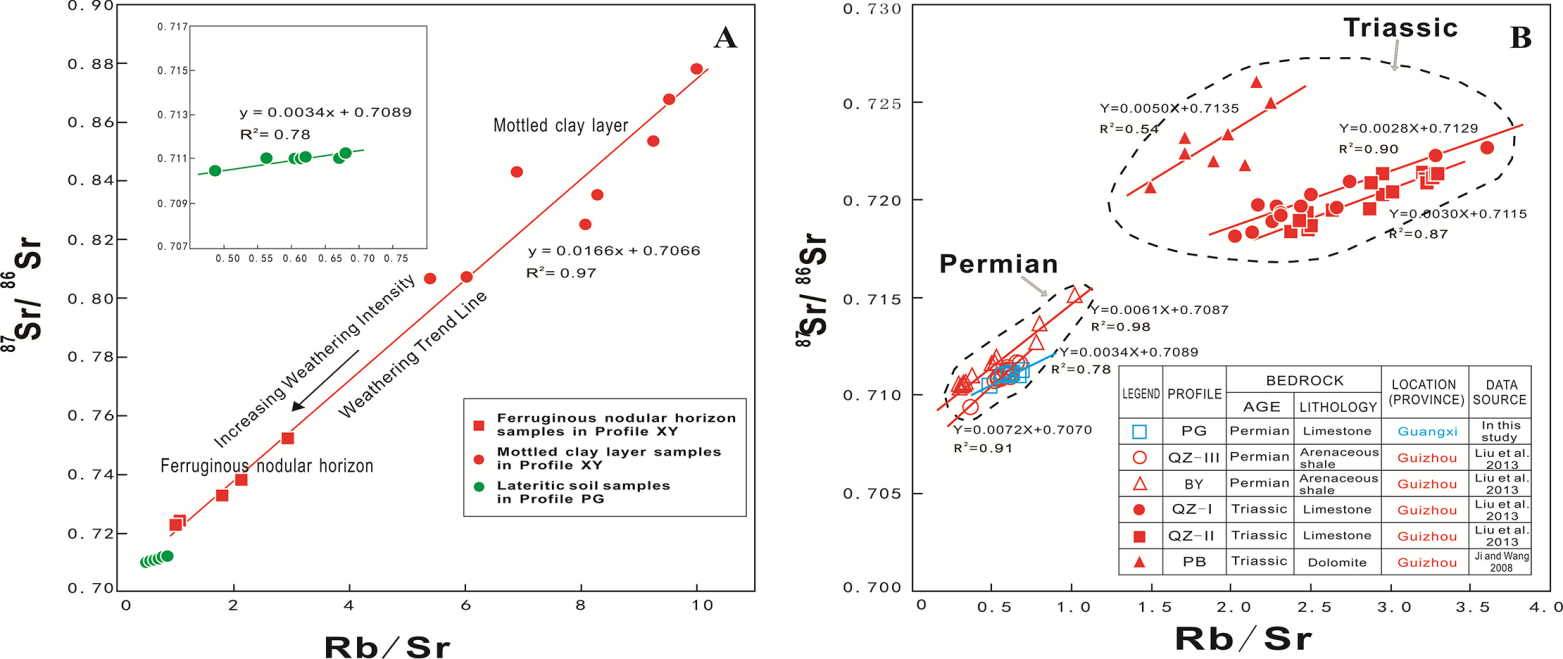
Relationships between ^87^Sr/^86^Sr and Rb/Sr ratios. (A) Weathering profiles in the studied area. (B) cited profiles in Guizhou province. (Data of profiles PB were derived from Ji and Wang [93]; data of profiles QZ–I, QZ–II, QZ–III and BY are from Liu et al. [62]).

In radioactive isotope chronology, the Rb–Sr isotope pair is commonly used to evaluate the crystallization age and sedimentary age of a closed rocky system [59, 85]. In the open supergene environment, however, weathering usually results in variations of the Rb and Sr concentrations and leaching rates of radiogenic and non-radiogenic Sr because of the diverse weathering rates of different minerals [43, 45–46, 61, 86–87]. Generally, minerals with high Rb/Sr ratios have high ^87^Sr/^86^Sr ratios and good resistance to weathering. If the radiogenic ^87^Sr in the weathering cover were primarily from the beta decay of ^87^Rb, then the weathering and decomposition of Rb-bearing minerals would lead to the loss of Rb and radiogenic ^87^Sr from the weathering system. The Rb/Sr and ^87^Sr/^86^Sr ratios of the two weathering profiles in the studied area show significant positive correlation (R^2^ = 0.97 in profile XY and R^2^ = 0.78 in profile PG) (Fig 8A). The strong linear relationships of the two weathering covers indicate radiogenic ^87^Sr is derived mainly from ^87^Rb decay, and the contribution from allothogenic ^87^Sr is very limited. In comparison with profile PG, the Rb/Sr and ^87^Sr/^86^Sr ratios of profile XY, especially in the mottled clay layer, are higher and spread over a larger interval, presumably because of the lesser degree of weathering and because the parent rock contained abundant minerals with high Rb/Sr ratios [88].

In geological history, the ^87^Sr/^86^Sr ratio of seawater was in dynamic equilibrium and therefore, the ^87^Sr/^86^Sr ratios of carbonate rocks deposited at that time should reflect the Sr isotope compositional signature of the seawater [43, 89–91]. Similarly, lateritic soils weathered from carbonate rocks could contain real geological information concerning the terrigenous detrital material of the source region [92].

**Fig 9:**
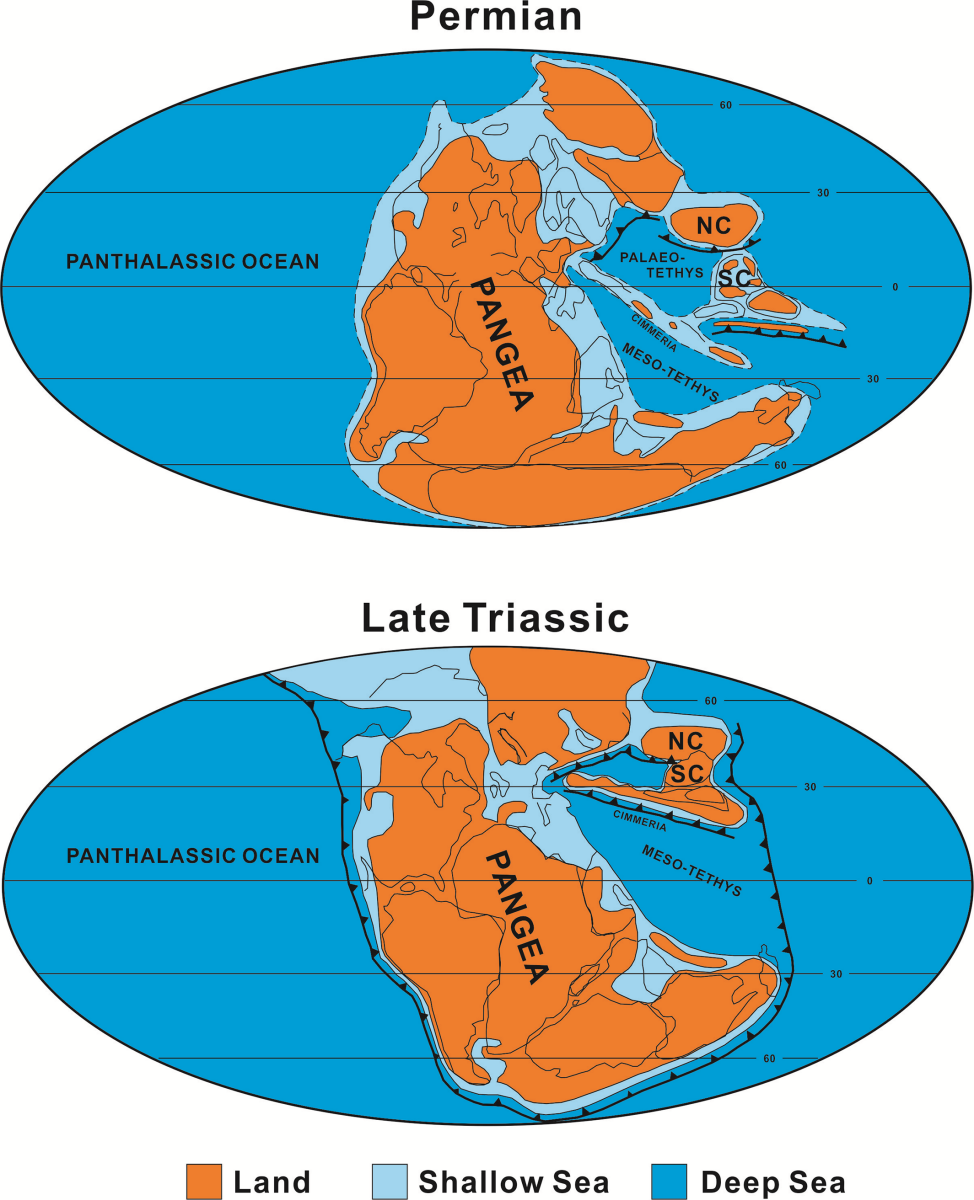
Global paleogeographic reconstruction in the Permian and the Late Triassic. NC: North China, SC: South China (after Scotese and Ziegler [96]).

To explore the Sr isotope source of weathering covers, the study profiles are compared with some analogous lateritic weathering profiles in Guizhou Province (Fig 8B). Some of the data are from published reports [62, 93] and the basic information of the weathering profiles is listed in the table in Fig 8B. In Fig 8B, lateritic soil samples are divided into two distinct groups according to the ages of the bedrock: Group T (bedrock is from the Triassic) and Group P (bedrock is from the Permian). In Group P, samples of profiles PG, QZ-III and BY are located in an adjacent area and they have similar intercepts for their regression equations (0.7070, 0.7087, and 0.7089, respectively). It indicates that, in the Permian, the area that contained Guangxi and Guizhou provinces (and possibly even a wider geographical area) was a big sedimentary basin with the same source of continental material. The situation is similar for Group T with the samples also having similar intercepts (0.7115, 0.7129, and 0.7135). However, Group T has higher Rb/Sr and ^87^Sr/^86^Sr ratios than Group P, implying that the continental fragment source of the South China sedimentary basin must have changed dramatically from the Permian to the Triassic. These changes of continental material source were possibly associated with plate tectonic activities. According to the lithofacies paleogeography, global plate arrangements during the Late Paleozoic were dominated by the supercontinent Pangea, bounded to the west by the Panthalassa Ocean and to the east by the tropical Tethys seaway (Fig 9). During the Late Permian, China consisted of several isolated small continents that existed in the Tethys seaway. During the late Triassic, the North China and South China blocks collided and migrated northward, until eventually colliding with Siberia (Fig 9) [94–95]. Therefore, the Sr isotopic records in lateritic soils likely provide evidence for the movement of the South China block during the Late Paleozoic.

Compared with profiles QZ–III and BY, profile PG presents a slightly lower ^87^Sr/^86^Sr ratio and a smaller slope of its regression equation (PG: 0.0034, BY: 0.0061, and QZ: 0.0072) (Fig 8B). The lower ^87^Sr/^86^Sr ratio suggests that the source area provided more radiogenic ^87^Sr terrigenous detrital material to the Guizhou area than to Guangxi. The smaller slope of the regression equation indicates that profile PG has experienced a higher degree of weathering than the other two profiles (QZ–III and BY). Generally, fine grain size fractions in weathering products represent highly weathered mineral facies, whereas coarse grain size fractions indicate weak weathering. Aubert et al. [44] proposed that the coarse grain size fractions of soils are more radiogenic than the fine grain size fractions. According to the principles of water transportation and deposition, coarse grain size materials are deposited preferentially. Thus, from the Sr isotopic composition of weathering covers in Guizhou and Guangxi provinces, we can infer that in the diagenetic period of the carbonate rocks (approximately from the Permian to the Middle Triassic), the Guizhou area was closer than the Guangxi area to the continental source. Relatively, the Guangxi region was further from the continental source and thus, it accepted epicontinental sea or hemipelagic deposition. The lithofacies paleogeography suggests that, in the Late Paleozoic, transgressions in the studied area always spread from south to north. The northern part of the Guizhou area was located in the Yangtze craton, whereas the Guangxi remained situated in the ancient sedimentary basin [97–99].

## Conclusions

An investigation was performed that used mineralogy, element geochemistry, and Sr isotope ratios to assess the Sr isotope geochemical characteristics and weathering processes of carbonate rock regolith. The results indicated that the mineral compositions and abundances in lateritic soils have an obvious effect on the Sr isotopic compositional signature. For example, weathering and decomposition of illite was found to be the primary factor that caused significant change of the Sr isotopic composition in profile XY that is an argillaceous limestone weathering crust with a lower weathering intensity. In addition, the major and trace element distributions in the vertical direction, REE distribution patterns, and Sr isotopic compositions showed that the accumulation of apatite, together with the adsorption and precipitation of Fe oxides/hydroxides, might possibly be another controlling factor of the Sr isotopic composition in the Fe nodular horizon. However, the weathering and dissolution of carbonate minerals (e.g., calcite and dolomite) in profile PG that is a limestone weathering crust with a higher weathering intensity were found to result in an increase of the ^87^Sr/^86^Sr ratio in the residual weathering products compared with the parent rock.

During the weathering and pedogenesis process of carbonate rocks, the leaching of carbonate minerals resulted in the increased ^87^Sr/^86^Sr ratio in the residual soils. When the carbonate components were leached out, the compositions and concentrations of silicate phases would largely dominated the ^87^Sr/^86^Sr ratio signatures. In addition, the proportional quantities of Fe nodules and clay are critical for controlling the Sr isotopic signature of the Fe nodular horizon in the typical lateritic weathering covers in Guangxi Province.

The results of this study suggest that parent rocks provided the main Sr isotopic source to the overlying weathering covers within the studied area, and that the contributions from allothogenic Sr isotopes were limited. The continental fragment source of the South China sedimentary basin changed dramatically from the Permian to the Triassic. This provides geochemical support for the movement of the South China block during the Late Paleozoic. In the Permian, the areas of Guizhou and Guangxi were located within the same sedimentary basin; however, the Guizhou area was closer to the continental source area, whereas the Guangxi region possibly accepted epicontinental sea or hemipelagic deposition.
